# Deubiquitinase USP16 induces gouty arthritis via Drp1-dependent mitochondrial fission and NLRP3 inflammasome activation

**DOI:** 10.1186/s13075-023-03095-7

**Published:** 2023-07-24

**Authors:** Qingdong Wang, Hongbin Qiu

**Affiliations:** Key Laboratory of Microecology-Immune Regulatory Network and Related Diseases, School of Basic Medicine, Jiamusi University, Heilongjiang Province, Jiamusi, 154000 People’s Republic of China

**Keywords:** USP16, Drp1, Mitochondrial fission, NF-κB, NLRP3, Gouty arthritis

## Abstract

**Background:**

Gouty arthritis is the most frequently diagnosed inflammatory arthritis worldwide. Dynamin-related protein 1 (Drp1), a regulator of mitochondrial fission, contributes to various inflammatory disorders via activating NLRP3 inflammasome. However, the biological role of Drp1 in gouty arthritis remains undefined.

**Methods:**

A mouse model of monosodium urate (MSU)-induced gouty arthritis and MSU-stimulated macrophages were established as in vivo and in vitro models, respectively. Histological changes were assessed by H&E and IHC analysis. RT-qPCR and western blot were used to detect the expression of Drp1 and the key molecules in joint tissues and macrophages. Cytokine secretion was measured by ELISA assay, and antioxidant enzymes activities and LDH release were monitored using commercial kits. Mitochondrial structure and functions were assessed by transmission electron microscopy (TEM) and MitoSOX staining. Co-IP and GST pull-down assay were used to detect the direct interaction between USP16 and Drp1, as well as the ubiquitination of Drp1.

**Results:**

Drp1 was elevated in MSU-induced gouty arthritis model, and it induced gouty arthritis via NF-κB pathway and NLRP3 inflammasome activation. In addition, Drp1 activated NF-κB/NLRP3 signaling via modulating mitochondrial fission. Mechanistically, USP16 mediated deubiquitination and stabilization of Drp1 through its direct interaction with Drp1. Functional studies further showed that USP16 was highly expressed in MSU-stimulated macrophages and induced gouty arthritis via Drp1-dependent NLRP3 inflammasome activation.

**Conclusion:**

Deubiquitinase USP16 induced gouty arthritis via Drp1-dependent mitochondrial fission and NF-κB/NLRP3 signaling.

**Supplementary Information:**

The online version contains supplementary material available at 10.1186/s13075-023-03095-7.

## Introduction

Gouty arthritis is the most frequently diagnosed inflammatory arthritis globally [1]. Higher prevalence of gout has been observed in China, and the rising incidence and prevalence of gout have been found by each decade of life [2]. The precipitation of monosodium urate (MSU) crystals within joints is a well-accepted cause of gouty arthritis. MSU crystals elicit inflammatory response which is principally mediated by macrophages and neutrophils [1, 2]. Compelling evidence has illustrated the key roles of NLRP3 inflammasome and the pro-inflammatory cytokine IL-1β in gouty arthritis [3]. MSU crystals are taken up by macrophages and trigger the NLRP3 inflammasome activation, as well as the maturation and release of IL-1β [4]. IL-1β further induces neutrophil articular infiltration, leading to joint pain [5]. In addition, the pro-oxidant, in particular mitochondrial reactive oxygen species (ROS), is also known to activate NLRP3 inflammasome [6, 7]. Identification of novel therapeutic target is needed to improve the clinical outcomes in gouty arthritis.

Dynamin-related protein 1 (Drp1) is recognized as the master regulator of mitochondrial fission [8]. In recent years, accumulating evidence has illustrated that Drp1 is implicated in the activation of NLPR3 inflammasome, and plays a crucial role in a variety of inflammatory disorders [9–12]. For instance, suppressing Drp1-dependent mitochondrial fission inhibits NLRP3 inflammasome activation in colitis-associated colorectal cancer (CRC) [12]. Mdivi-1, an inhibitor of Drp1, alleviates acute kidney injury (AKI) by suppressing NLRP3 inflammasome activation [11]. Interestingly, Mdivi-1 or knockdown of Drp1 has been reported to suppress NF-κB signaling in macrophage/microglial cells [13, 14]. It is well-established that NF-κB signaling regulates NLRP3 inflammasome in macrophages [15], raising the possibility that Drp1 might activate NLRP3 inflammasome though NF-κB signaling. However, it remains uninvestigated whether Drp1-dependent mitochondrial fission and NLRP3 inflammasome activation are involved in gouty arthritis.

Ubiquitination, one of the most important post-translational modification of proteins, regulates various physiological and pathological processes [16]. Deubiquitinases (DUBs) remove ubiquitin signals and have been linked with different diseases, including neurodegenerative disorders, cancer and inflammatory diseases [17]. Ubiquitin specific peptidase 16 (USP16) is originally identified as a DUB of histone H2A which is implicated in cell cycle progression and HOXD10 gene expression [18]. Knockout study has reported that depletion of USP16 results in early embryonic lethality in mice [19]. However, the biological function of USP16 in gouty arthritis remains elusive. A recent study has demonstrated that USP16 promotes autoimmune inflammation by mediating IKKβ deubiquitination [20]. Our bioinformatics analysis and preliminary data predicted that USP16 might act as a DUB of Drp1. We thus hypothesized that USP16 might contribute to gouty arthritis via Drp1-dependent mitochondrial fission and NLRP3 inflammasome activation.

In this study, we reported that Drp1 was elevated in MSU-induced gouty arthritis model, and it induced gouty arthritis via NF-κB pathway and NLRP3 inflammasome activation. By monitoring the mitochondrial structure and function, we found that Drp1 activated NF-κB/NLRP3 signaling via modulating mitochondrial fission. USP16 mediated deubiquitination and stabilization of Drp1 through its direct interaction with Drp1. Functional studies further showed that USP16 was highly expressed in MSU-stimulated macrophages and induced gouty arthritis via Drp1-dependent NLRP3 inflammasome activation.

## Materials and methods

### Clinical specimens

Synovial tissues from patients with gouty arthritis (*n* = 4) and patients with acute cruciate ligament injury (control, *n* = 4) were collected by arthroscopy as described [21]. This study was approved by the Affiliated First Hospital of Jiamusi University. Written consents were obtained from all patients.

### Animal study

C57BL/6 mice (6–8-weeks-old, *n* = 6 per group) were from Key laboratory of Microecology-immune Regulatory Network and Related Diseases, School of Basic Medicine, Jiamusi University. All animal studies were approved by Key laboratory of Microecology-immune Regulatory Network and Related Diseases, School of Basic Medicine, Jiamusi University. Monosodium urate (MSU)-induced gouty arthritis model was established as previously described [22]. MSU (0.5 mg dissolved in 20 μL sterile PBS) was administrated by intraarticular injection. After 24 h, the patella was harvested. For Drp1 knockdown study, sh-Drp1 construct was injected intraarticularly 1 h prior to MSU injection.

### Cell culture, transfection and treatment

Mouse macrophages RAW264.7, J774A.1 cells and human embryonic kidney (HEK293T) cells were obtained from ATCC (Manassas, VA, USA). Cells were grown in RPMI1640 containing 10% FBS (Gibco, Grand Island, NY, USA), and maintained in a CO_2_ incubator at 37 °C. sh-NC, sh-Drp1 and sh-USP16 were obtained from GenePharma (Shanghai, China). The full-length of Drp1, USP7, USP8, USP10, USP13, USP15, USP16, USP25, USP28, USP 30, USP32, USP42, USP48 were cloned into pcDNA3.1 vector (Invitrogen, Carlsbad, CA, USA). For MSU-induced in vitro model, RAW264.7 cells were transfected with sh-NC, sh-Drp1 or sh-USP16 and/or overexpression construct (400 ng/μL) using Lipofectamine 3000 (Invitrogen). sh-Drp1-2 and sh-USP16-3 were selected for the subsequent experiments, and the other shRNAs were found to be less effective in silencing Drp1 or USP16. At 48 h post-transfection, cells were stimulated with LPS (100 ng/mL) for 1 h, followed by the incubation with MSU (200 μg/mL) for 6 h. For cell treatment, macrophages were treated with BAY 11–7082 (4 μM), CHX (5 μg/mL), MG132 (20 μM) or mitoTEMPOL (20 μM) for specific period of time. All the chemicals except mitoTEMPOL (ab144644, Abcam, Cambridge, UK) were purchased from Sigma-Aldrich (St. Louis, MO, USA).

### Histological analysis

The collected ankle joint tissues were fix in 4% paraformaldehyde (PFA) and decalcified for 14 days. The tissues were embedded in paraffin and subjected to hematoxylin & eosin (H&E) staining as described [23]. For immunohistochemistry (IHC), the sections were subjected to antigen retrieval and permeabilization. After blocking, the slides were incubated with anti-Drp1 antibody (1:200, ab184247, Abcam) at 4 °C overnight. This was followed by the incubation with goat anti-rabbit IgG-HRP, and detected using DAB substrate.

### RT-qPCR

Total RNA was extracted using Trizol reagent (Invitrogen), and reverse transcribed using PrimeScript RT reagent (TaKaRa, Dalian, China). RT-qPCR was performed using a SYBR Green MasterMix (Invitrogen). The results were calculated using 2 ^–ΔΔCT^ method. GAPDH served as an internal control. Primers used in RT-qPCR were listed in Table 1.

**Table 1:** The primer used in the manuscript

Primer	Sequence 5’-3’
Drp1 sense	ATGCCTGTGGGCTAATGAAC
Drp1 anti-sense	AGTTGCCTGTTGTTGGTTCC
USP16 sense	CCCGGAATGAGAAACTTCAA
USP16 anti-sense	AGCATCTGCTTCTTGGCATT
GAPDH sense	AGCCCAAGATGCCCTTCAGT
GAPDH anti-sense	CCGTGTTCCTACCCCCAATG

### Western blot

Protein lysates were extracted using IP lysis buffer supplemented with protease inhibitor cocktail (Pierce, Rockford, IL, USA). Equal amounts of proteins were separated by SDS-PAGE, and transferred onto NC membranes. After blocking, the blots were incubated with primary antibody at 4 °C overnight, followed by the incubation with corresponding secondary antibody. The signal was detected using ECL detection reagent (Pierce). The antibodies used in RT-qPCR were listed in Table 2.

**Table 2:** The Antibodies used in the manuscript

Antibody	Vendor	Catalog no	Working dilution
Drp1	Abcam	ab184247	WB (1:1000); IP (1 μg); IHC (1:200)
p65	CST	#8242	WB (1:1000)
p-p65	CST	#3033	WB (1:1000)
IκBα	CST	#4814	WB (1:1000)
p-IκBα	CST	#2859	WB (1:1000)
NLRP3	Abcam	ab263899	WB (1:1000)
Caspase-1	Abcam	ab1384832	WB (1:1000)
USP16	Proteintech	14,055–1-AP	WB (1:1000); IP (1 μg)
GAPDH	Abcam	ab8245	WB (1:2000)

Abcam, Cambridge, UK. Cell Signaling Technology (CST), Danvers, MA, USA; Proteintech, Chicago, IL, USA

### ELISA assay

The levels of IL-1β, TNF-α and IL-6 in tissue homogenates or cell culture supernatant were determined by ELISA assay. IL-1β (BMS6002, Invitogen), TNF-α (BMS607-3, Invitogen) and IL-6 (BMS603-2, Invitogen) mouse ELISA kits. ELISA assay was conducted according to the manufacturer’s instructions. A450 was measured by a microplate reader (Thermo Fisher Scientific).

### Measurement of the antioxidant enzyme activities

The activities of CAT and GSH-Px were detected by Catalase Activity Assay Kit (ab83464, Abcam) and Glutathione Peroxidase Assay Kit (ab102530, Abcam), respectively. SOD activity was assessed by Superoxide Dismutase Activity Assay Kit (ab65354, Abcam). The assays were performed according to the manufacturer’s protocols.

### LDH release assay

LDA release was monitored using CyQUANT LDH Cytotoxicity Assay (C20300, Invitrogen) according to the manufacturer’s instructions. A490 and A680 were measured using a microplate reader. A680 served as a reference wavelength.

### Transmission electron microscopy (TEM)

Cells were fixed with 4% glutaraldehyde. After dehydration and araldite embedding, the samples were incubated with 1% osmium tetroxide. The sections were then stained with uranyl acetate and lead citrate, and observed using a TEM (Thermo Fisher Scientific).

### Protein stability assay

Protein stability assay was performed as described [24]. RAW264.7 or J774A.1 cells were treated with CHX (5 μg/mL, Sigma-Aldrich) for 0, 15, 30, 60, 120 and 240 min. Cells were collected and lysed at different timepoints, followed by western blot analysis. The results were normalized and quantified using Quantity One Software (Bio-Rad).

### Mitochondrial superoxide measurement

Mitochondrial superoxide was determined by using MitoSOX red (M36008, Invitrogen), respectively. In brief, RAW264.7 or J774A.1 cells were incubated with 5 μM MitoSOX Red at 37 °C for 30 min. Images were acquired using confocal microscopy (Nikon, Tokyo, Japan).

### Co-immunoprecipitation (Co-IP)

Co-IP was conducted using Pierce Crosslink Magnetic co-IP Kit (Pierce). Briefly, cell lysates were prepared using IP lysis buffer and incubated with anti-USP16 (1 μg, 14,055–1-AP, Proteintech, Chicago, IL, USA), anti- or normal IgG-conjugated Protein A/G beads at 4 °C overnight. The immunoprecipitated protein complex were then eluted and analyzed by western blot. Whole cell lysates were used as an input control.

### GST Pull-down assay

GST Pull-down assay was conducted using Pierce GST Pull-Down Kit (Pierce). In brief, the plasmids for GST-USP16 were transfected into E. coli. The fusion of 5 × 10^4^ RAW264.7 or J774A.1 cells per well and cultured in medium with 0.1% bovine serum albumin for 24 h. GST tag alone or GST-tagged USP16 was immobilized to glutathione agarose. The cell lysates were then incubated with agarose. The enriched proteins were eluted and analyzed by western blot. Whole cell lysates were used as an input control.

### Statistical analysis

Data are expressed as the mean ± S.D. Statistical analysis was performed by one-way ANOVA or Student’s t-test. Statistical analysis was performed using SPSS 22.0 (SPSS, Chicago, IL, USA). Significance was set at *, *P* < 0.05.

## Results

### Drp1 induces gouty arthritis

**Fig. 1:**
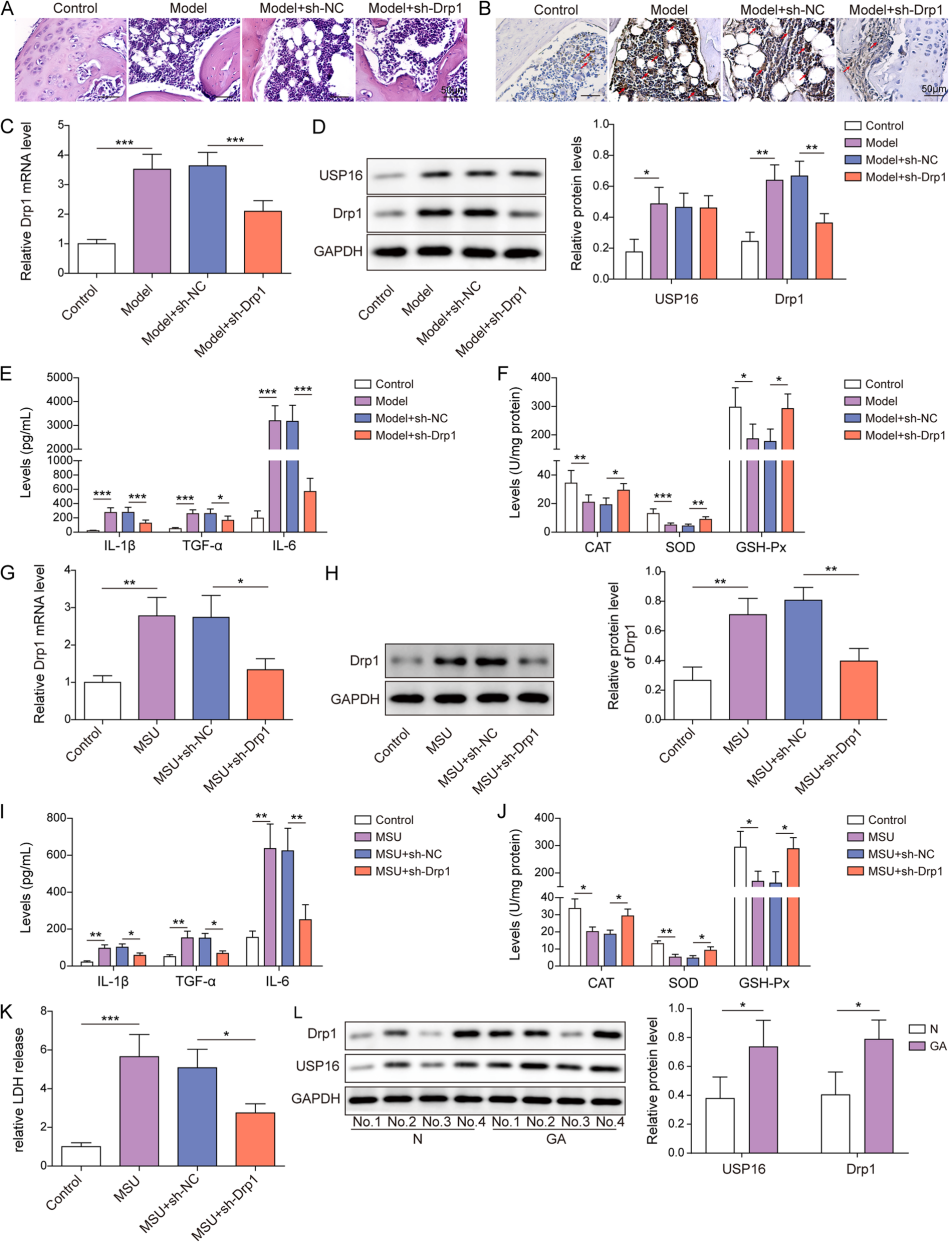
Drp1 induces gouty arthritis. **A** The histological changes in the joints were assessed by H&E staining. Scale bar = 50 μm. **B** The immunoreactivity of Drp1 in the joints was detected by IHC. Scale bar = 50 μm. **C**, **F** The mRNA level of Drp1 was detected by RT-qPCR. **D** The protein levels of Drp1 and USP16 in the joints were detected by western blot. **E**, **I** The secretion of IL-1β, TNF-α and IL-6 were measured by ELISA assay. **F**, **J** The activities of antioxidant enzymes were detected using commercial kits. **H** The protein level of Drp1 was detected by western blot. **K** The LDH release was measured using commercial kit. **L** The protein levels of Drp1 and USP16 in the synovial tissues were detected by western blot. *, *P* < 0.05, **, *P* < 0.01, ***, *P* < 0.001

To explore the biological roles of Drp1 in gouty arthritis, a mouse model of MSU-induced gouty arthritis was established. As presented in Fig. 1A, H&E staining showed that MSU-induced leukocyte infiltration, synovial thickening, vacuole formation and interstitial edema were effectively reduced by Drp1 knockdown. IHC coupled with RT-qPCR revealed that Drp1 was dramatically elevated in MSU-treated mice, and in vivo transfection of sh-Drp1 successfully decreased Drp1 level in the joint cartilages (Fig. 1B-C). The knockdown efficiency of sh-Drp1 in RAW264.7 and J774A.1 cells was detected by western blot, and sh-Drp1-2 with high knockdown efficiency was selected for the subsequent experiment (Fig. S1A). Consistently, western blot showed that Drp1 and USP16 were remarkably increased in the joints of mice with gouty arthritis. Silencing of Drp1 abolished MSU-induced Drp1 expression, while it had no effect on USP16 expression, suggesting that USP16 might act as an upstream molecule of Drp1 (Fig. 1D). ELISA assay showed that MSU-increased secretion of IL-1β, TNF-α and IL-6 in the joints were reversed by sh-Drp1 (Fig. 1E). In contrast, MSU-decreased activities of antioxidant enzymes, namely CAT, GSH-Px and SOD, were rescued by Drp1 silencing (Fig. 1F). We next sought to validate these findings in the in vitro model. Drp1 expression was much higher in MSU-treated RAW264.7 cells than that in control cells, and silencing of Drp1 partially attenuated MSU-mediated upregulation of Drp1 at both mRNA and protein levels (Fig. 1G-H). In agreement with the in vivo findings, MSU-induced IL-1β, TNF-α and IL-6 in cell culture supernatant were abrogated by Drp1 knockdown (Fig. 1I). Silencing of Drp1 also reversed MSU-mediated changes of antioxidant enzymes or LDH release in RAW264.7 cells (Fig. 1J-K). As expected, Drp1 and USP16 were elevated in the synovial tissues from patients with gouty arthritis, compared with control group (Fig. 1L). These data clearly suggest that Drp1 contributes to gouty arthritis in vivo and in vitro.

### Drp1 induces gouty arthritis via NF-κB pathway and NLRP3 inflammasome activation

**Fig. 2:**
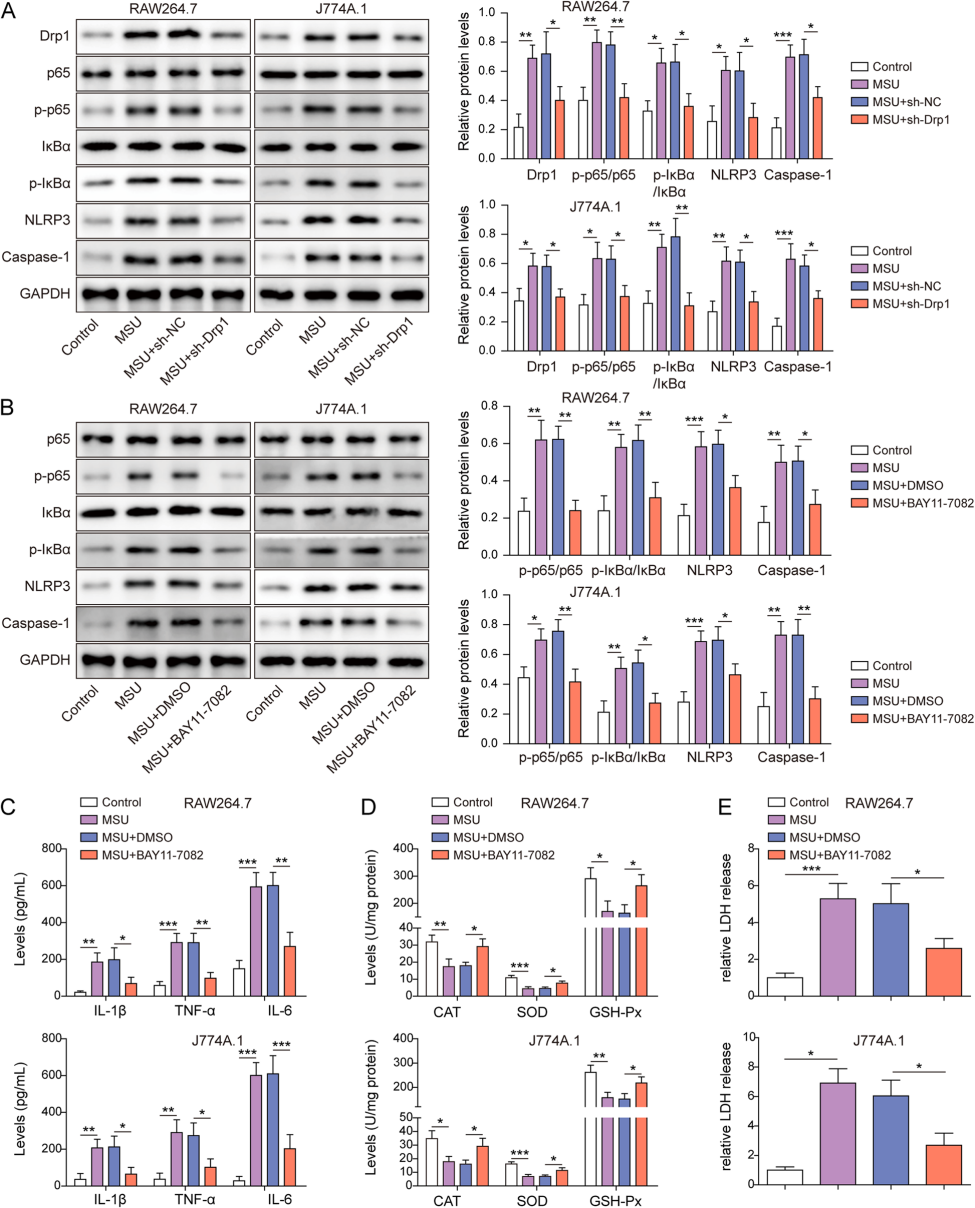
Drp1 induces gouty arthritis via NF-κB pathway and NLRP3 inflammasome activation. **A** The protein levels of Drp1 and key molecules of NF-κB/NLRP3 signaling were detected by western blot. **B**, The protein levels of key molecules of NF-κB/NLRP3 signaling were detected by western blot. **C** The secretion of IL-1β, TNF-α and IL-6 were measured by ELISA assay. **D** The activities of antioxidant enzymes were detected using commercial kits. **E** The LDH release was measured using commercial kit. *, *P* < 0.05, **, *P* < 0.01, ***, *P* < 0.001

We next investigated the downstream signalings of Drp1 in macrophages. Western blot showed that MSU activated NF-κB signaling and NLRP3 inflammasome in RAW264.7 and J774A.1 cells in which the levels of p-p65, p-IκBα, NLRP3 and Caspase-1 were upregulated by MSU (Fig. 2A). Knockdown of Drp1 abolished the effects of MSU on the expression of these proteins (Fig. 2A). Intriguingly, the NF-κB inhibitor BAY 11–7082 and sh-Drp1 exerted similar effects in RAW264.7 and J774A.1 cells (Fig. 2B). Moreover, MSU-induced secretion of IL-1β, TNF-α and IL-6 were abrogated by BAY 11–7082 (Fig. 2C). BAY 11–7082 also rescued MSU-suppressed activities of CAT, GSH-Px and SOD in RAW264.7 and J774A.1 cells (Fig. 2D). Furthermore, the release of LDH from macrophages in response to MSU was reversed by BAY 11–7082 (Fig. 2E). These findings suggest that NF-κB signaling and NLRP3 inflammasome play critical roles in Drp1-induced gouty arthritis.

### Drp1 activates NF-κB pathway and NLRP3 inflammasome by modulating mitochondrial fission

**Fig. 3:**
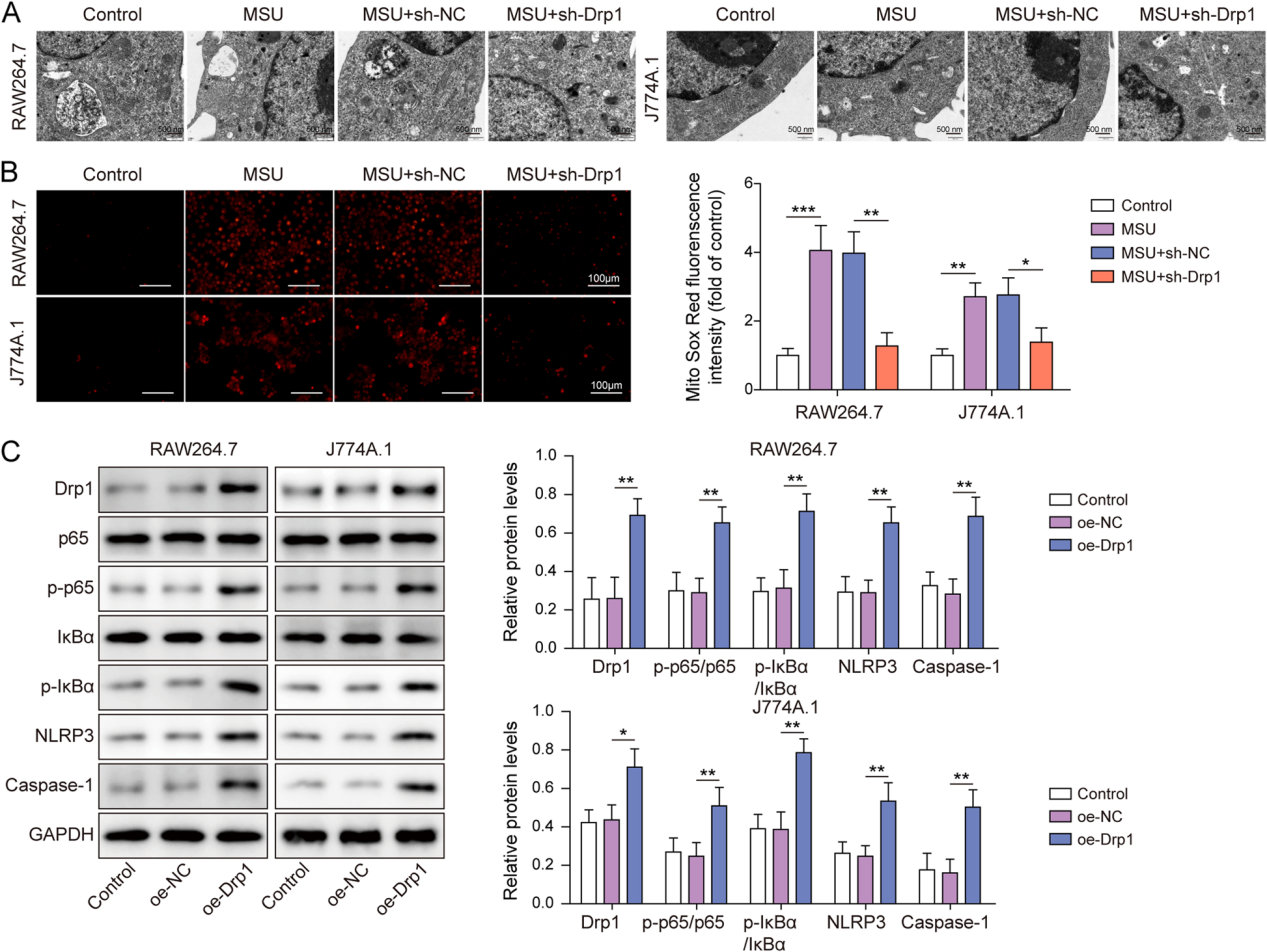
Drp1 activates NF-κB pathway and NLRP3 inflammasome by modulating mitochondrial fission. **A** TEM analysis of mitochondria from RAW264.7 and J774A.1 cells. Scale bar = 500 nm. **B** Mitochondrial ROS was assessed by MitoROX staining. Red, MitoROX. Scale bar = 100 μm. **C** The protein levels of Drp1 and key molecules of NF-κB/NLRP3 signaling were detected by western blot. *, *P* < 0.05, **, *P* < 0.01, ***, *P* < 0.001

Mitochondria dysfunction has been observed in MSU-stimulated macrophages [25]. To unravel the role of Drp1 in MSU-induced mitochondrial damage, TEM was employed to examine the mitochondrial structure. As presented in Fig. 3A, mitochondria in control cells exhibited intact outer membrane and densely packed cristae, whereas MSU treatment resulted in dilated mitochondria with swelling or disrupted cristae. Knockdown of Drp1 alleviated MSU-disrupted mitochondrial structure (Fig. 3A). Additionally, MitoSOX staining further revealed that MSU-induced mitochondrial ROS were attenuated in Drp1-knockdown macrophages (Fig. 3B). We next examined the effects of Drp1 on the expression of the key molecules of NF-κB signaling and NLRP3 inflammasome. Transfection of Drp1 overexpression construct increased Drp1 protein level dose-dependently, and RAW264.7 and J774A.1 cells were transfected with 400 ng/μL plasmid in the subsequent experiments (Fig. S1B). Western blot showed that overexpression of Drp1 successfully increased Drp1 protein level, as well as the expression of p-p65, p-IκBα, NLRP3 and Caspase-1 in both RAW264.7 and J774A.1 cells (Fig. 3C). In addition, Drp1-induced p-p65, p-IκBα, NLRP3 and Caspase-1 were further attenuated by NF-κB inhibitor BAY 11–7082 (Fig. S2), indicating the pivotal role of NF-κB signaling in Drp1-regulated mitochondrial fission and NLRP3 inflammation. Moreover, Drp1-upragulated NLRP3 and Caspase-1 were counteracted by MitoTEMPOL in both RAW264.7 and J774A.1 cells (Fig. S3A). Similar results were also observed in MSU-simulated macrophages (Fig. S3B), indicating that mitochondrial ROS responsible for augmenting mitochondrial fission contributes to Drp1-induced NLPR3 inflammasome activation. Collectively, these data indicate that Drp1-dependent mitochondrial fission is associated with the activation of NF-κB pathway and NLRP3 inflammasome.

### Deubiquitinase USP16 directly interacts with Drp1

**Fig. 4:**
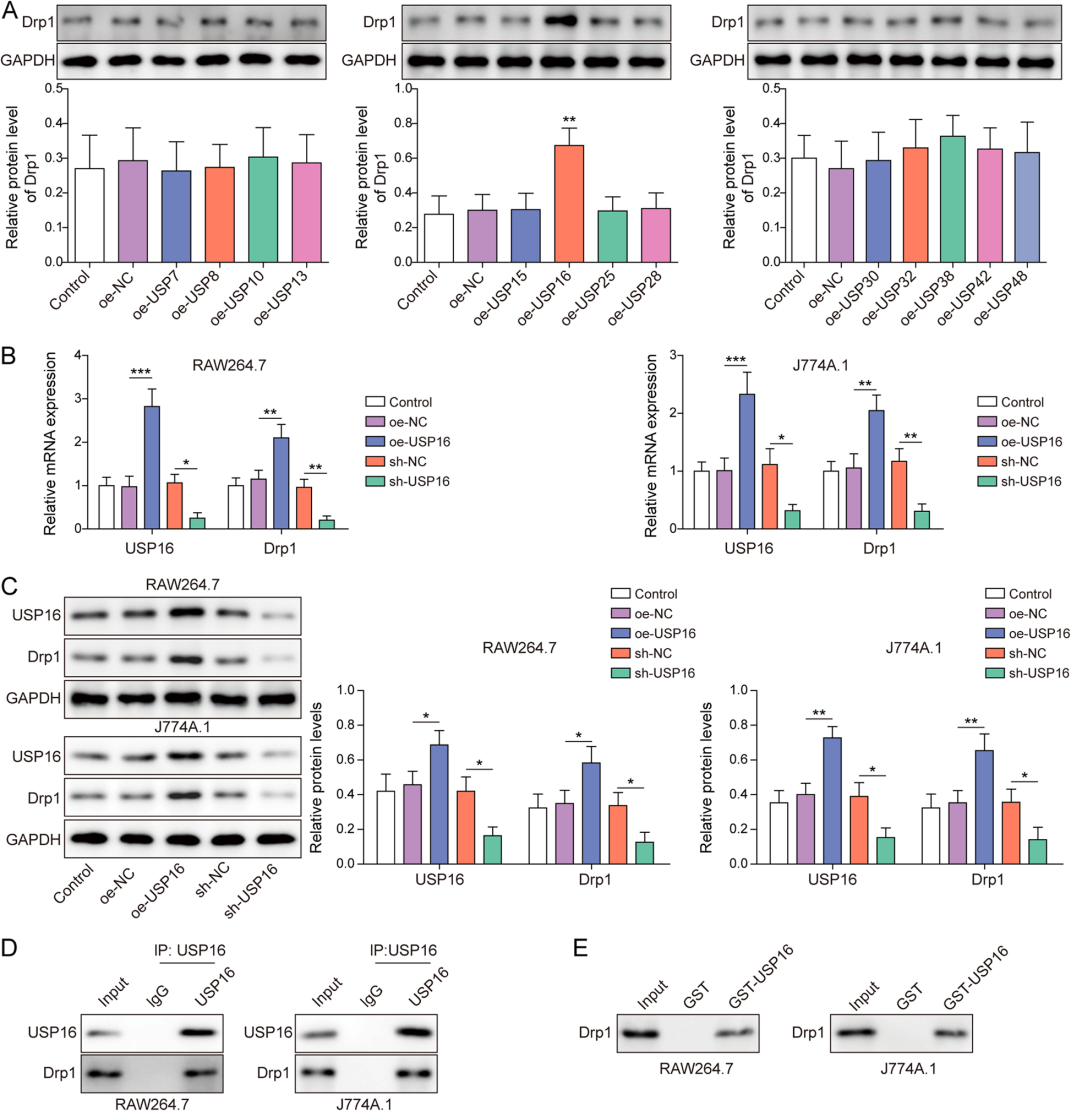
Deubiquitinase USP16 directly interacts with Drp1. **A** The protein level of Drp1 was detected by western blot. The mRNA (**B**) or protein levels (**C**) of USP16 and Drp1 were detected by RT-qPCR or western blot, respectively. **D** The direct interaction between USP16 and Drp1 in macrophages was assessed by Co-IP. Whole cell lysates were used as input control. Normal IgG served as a negative control. **E** The in vitro interaction between GST-tagged USP16 and Drp1 was detected by GST pull-down assay. Whole cell lysates were used as input control. Vector alone acted as a negative control. *, *P* < 0.05, **, *P* < 0.01, ***, *P* < 0.001

We next sought to screen the UbiBrowser-predicted putative deubiquitinases of Drp1 by western blot. Among 13 deubiquitinases, the results of western blot showed that USP16 overexpression dramatically upregulated Drp1 expression in HEK293T cells. By contrast, the other deubiquitinases, including USP7, USP8, USP10, USP13, USP15, USP25, USP28, USP30, USP32, USP38, USP42 and USP48, had no significant effect on Drp1 protein level (Fig. 4A). In accordance with this finding, overexpression and knockdown experiments revealed that USP16 positively regulated Drp1 mRNA and protein levels in both RAW264.7 and J774A.1 cells (Fig. 4B-C). The knockdown efficiency of sh-USP16 was detected by western blot, and shUSP16-3 with high silencing efficiency was selected for the subsequent experiments (Fig. S1C). Co-IP showed that antibody against USP16 successfully immunoprecipitated Drp1 in macrophages (Fig. 4D). This result was further validated by GST pull-down assay in which GST-tagged USP16 enriched Drp1 in vitro. Taken together, these data suggest the direct association between USP16 and Drp1, and USP16 positively regulated Drp1 expression in RAW264.7 and J774A.1 cells.

### Deubiquitination and stabilization of Drp1 by USP16

**Fig. 5:**
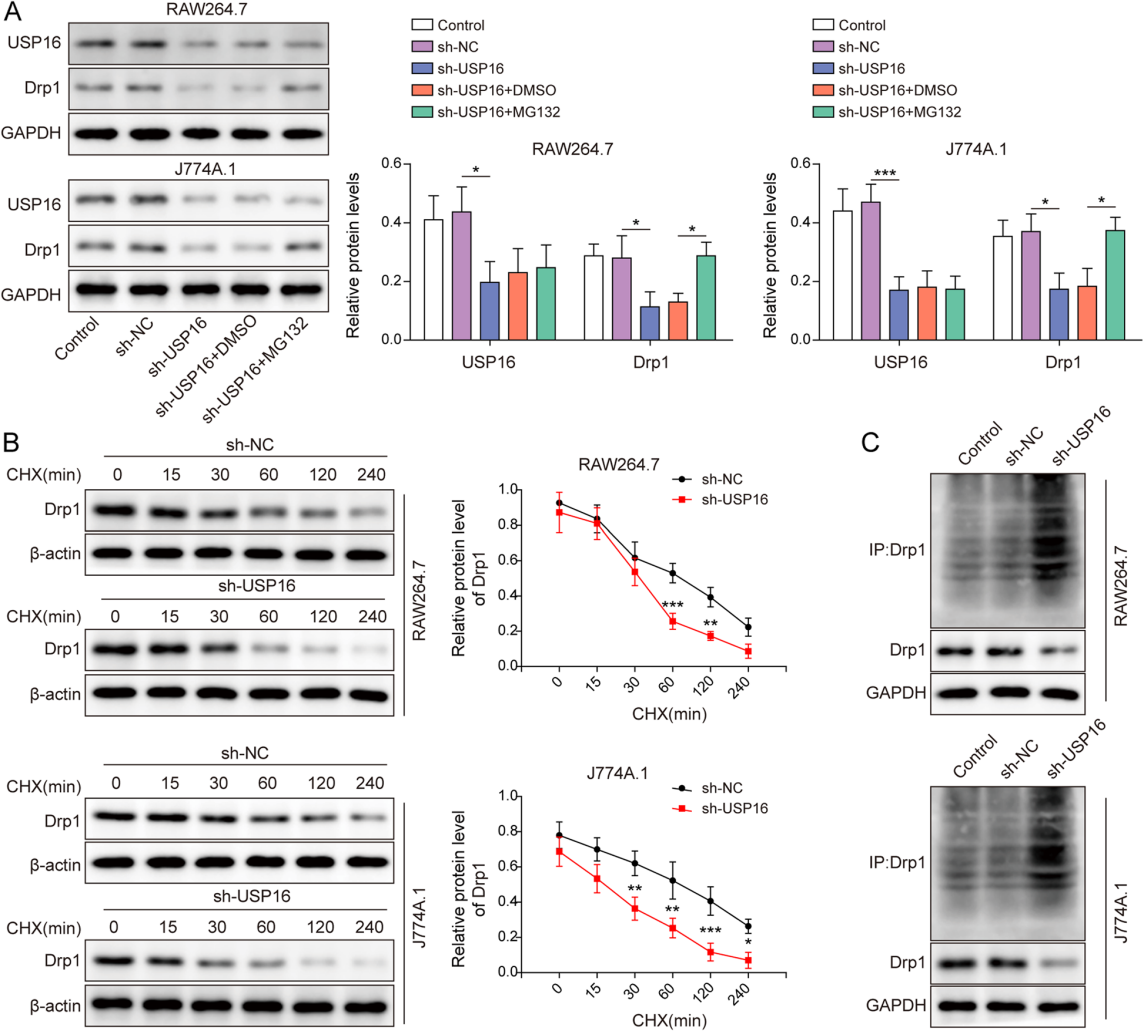
Deubiquitination and stabilization of Drp1 by USP16. **A** The protein levels of USP16 and Drp1 in macrophages were detected by western blot. **B** The protein stability of Drp1 was examined by western blot in the presence of CHX. **C** The ubiquitination of Drp1 was assessed by Co-IP. GAPDH acted as an internal control. *, *P* < 0.05, **, *P* < 0.01, ***, *P* < 0.001

To investigate the function of USP16, we next test whether USP16 was implicated in the deubiquitination and stabilization of Drp1. As anticipated, silencing of USP16 caused a reduction of Drp1, whereas the proteasome inhibitor MG132 led to a rebound of Drp1 in both RAW264.7 and J774A.1 cells (Fig. 5A), indicating that lack of USP16 promoted Drp1 turnover via ubiquitin–proteasome pathway. Moreover, USP16 knockdown promoted the degradation of Drp1 in the presence of protein synthesis inhibitor CHX (Fig. 5B). Furthermore, Co-IP showed that silencing of USP16 remarkably increased the ubiquitination of Drp1 in the presence of MG132 (Fig. 5C). These findings suggest that USP16 mediates deubiquitination and stabilization of Drp1 in RAW264.7 and J774A.1 cells.

### USP16 is elevated and contributes to gouty arthritis in vitro

**Fig. 6:**
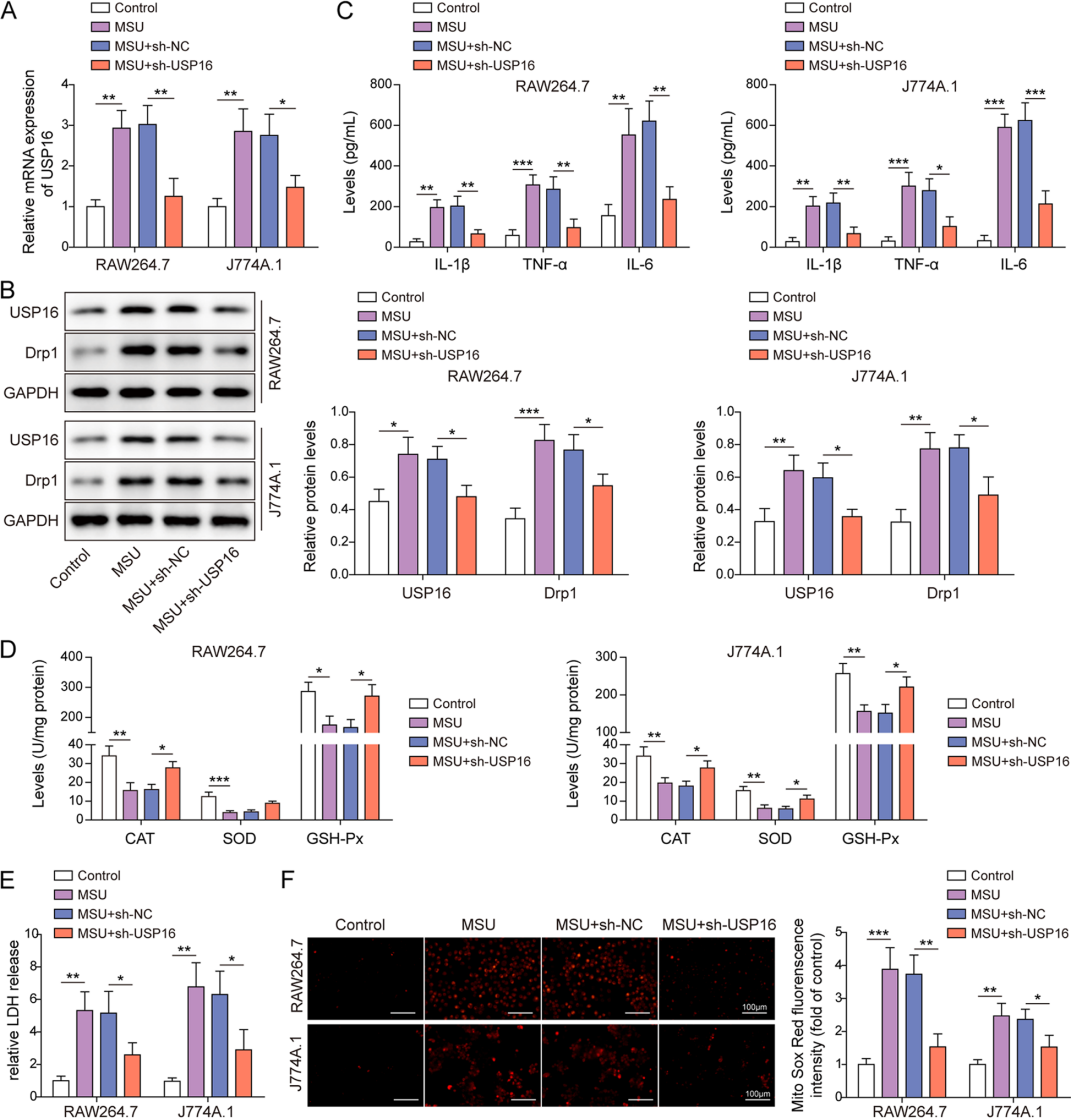
USP16 is elevated and contributes to gouty arthritis in vitro. **A** The mRNA level of USP16 was detected by RT-qPCR. **B** The protein levels of USP16 and Drp1 in RAW264.7 and J774A.1 cells were detected by western blot. **C** The secretion of IL-1β, TNF-α and IL-6 were measured by ELISA assay. **D** The activities of antioxidant enzymes were detected using commercial kits. **E** The LDH release was measured using commercial kit. **F** Mitochondrial ROS was assessed by MitoROX staining. Red, MitoROX. Scale bar = 100 μm. *, *P* < 0.05, **, *P* < 0.01, ***, *P* < 0.001

To delineate the biological roles of USP16, the expression and functions of USP16 were examined in MSU-stimulated macrophages. As show in Fig. 6A-B, the expression of USP16 and Drp1 were greatly increased upon MSU treatment, and knockdown of USP16 reduced USP16 expression, alone with the reduction of Drp1 in both RAW264.7 and J774A.1 cells. ELISA assay revealed that silencing of USP16 counteracted MSU-induced secretion of IL-1β, TNF-α and IL-6 in macrophages (Fig. 6C). Depletion of USP16 also reversed MSU-mediated changes of antioxidant enzymes activities and LDH release (Fig. 6D-E). In addition, MSU-induced mitochondrial ROS were abrogated by USP16 knockdown in RAW264.7 and J774A.1 cells (Fig. 6F). Together, the functional studies indicate that USP16 might act as a key player in gouty arthritis.

### USP16 induces gouty arthritis via Drp1-dependent NLRP3 inflammasome activation

**Fig. 7:**
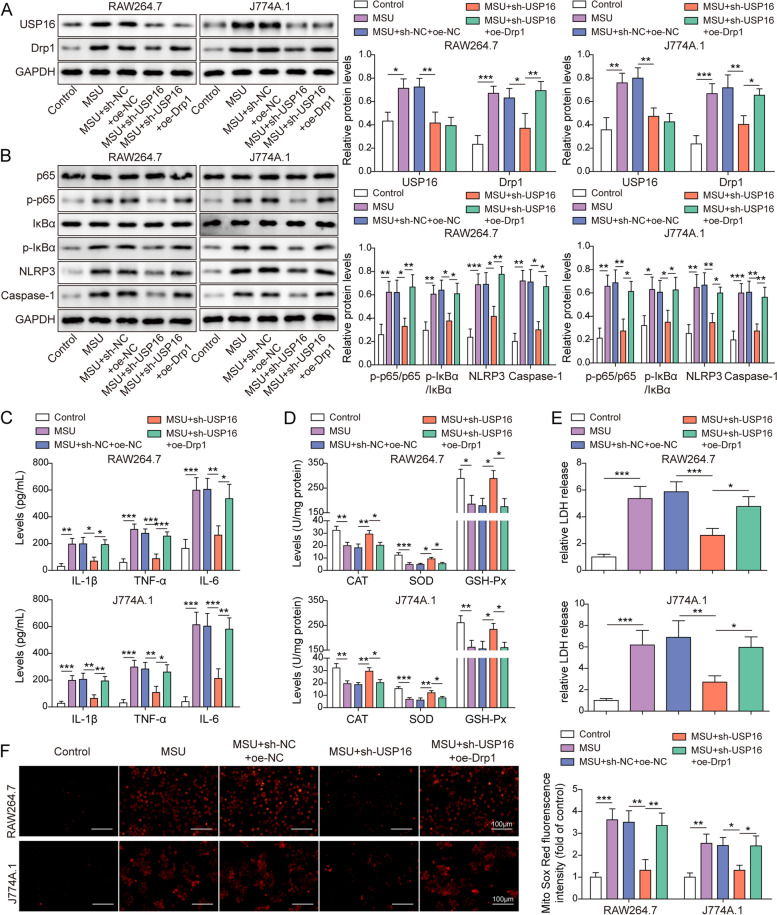
USP16 induces gouty arthritis via Drp1-dependent NLRP3 inflammasome activation. **A** The protein levels of USP16 and Drp1 was detected by western blot. **B** The protein levels of the key molecules of NF-κB/NLRP3 signaling were detected by western blot. **C** The secretion of IL-1β, TNF-α and IL-6 were measured by ELISA assay. **D** The activities of antioxidant enzymes were detected using commercial kits. **E** The LDH release was measured using commercial kit. **F** Mitochondrial ROS was assessed by MitoROX staining. Red, MitoROX. Scale bar = 100 μm. *, *P* < 0.05, **, *P* < 0.01, ***, *P* < 0.001

We further tested whether Drp1 functioned as a downstream effector of USP16 in MSU-stimulated macrophages. Western blot revealed that knockdown of USP16 reversed MSU-mediated up-regulation of USP16 and Drp1, whereas the effect of sh-USP16 on Drp1 expression was counteracted by Drp1 overexpression. It is worth noting that overexpression of Drp1 had no effect on USP16 expression at protein levels (Fig. 7A). Interestingly, silencing of USP16 blocked MSU-activated NF-κB pathway and NLRP3 inflammasome, while Drp1 overexpression reversed these effects of sh-USP16 in RAW264.7 and J774A.1 cells as detected by western blot (Fig. 7B). In addition, MSU-induced secretion of IL-1β, TNF-α and IL-6 were abolished by sh-USP16, and Drp1 overexpression resulted in a rebound of these cytokine levels (Fig. 7C). Similarly, the rescue effects of sh-USP16 on antioxidant enzymes activities, LDH release and mitochondrial ROS were also reversed by Drp1 overexpression (Fig. 7D-F). These findings suggest that USP16 induces gouty arthritis via Drp1-dependent NLRP3 inflammasome activation.

## Discussion

Gout arthritis results from hyperuricemia, leading to deposition of MSU crystal within the joints affecting synovial membrane and articular cartilage [1]. Patients experience acute episodes of joint pain which is usually interspersed with symptom-free periods. Left untreated, gout arthritis typically progress to crystal aggregates in soft tissues, thus destroying cartilage and bone [26, 27]. In the current study, we reported that USP16 induced gout arthritis via mediating deubiquitination and stabilization of Drp1. Mechanistically, Drp1-dependent mitochondrial fission and NF-κB/NLRP3 signaling play indispensable roles during this process.

Drp1, a GTPase, regulates mitochondrial fission by oligomerization on the outer mitochondrial membrane [28]. Previous studies in myocardial ischemia–reperfusion (I/R) injury or hemorrhagic shock have illustrated that phosphorylation of Drp1 causes increased mitochondrial ROS production [29, 30]. Emerging evidence suggests that mitochondrial ROS fuels NLRP3 inflammasome [7, 31, 32]. Upon stimulation, NLRP3 inflammasomes are assembled by recruiting the adaptor ASC and Caspase-1, thereby proteolytic processing IL-1β and IL-18 [33]. In gout arthritis, IL-1β triggers an inflammatory response which characterized by neutrophils infiltration and vasodilatation [34]. In line with these findings, our data revealed that lack of Drp1 alleviated MSU-induced gout arthritis, alone with the reduction of mitochondrial ROS. In addition, Drp1 knockdown and BAY 11–7082 exerted similar effects on NF-κB/NLRP3 signaling molecules, indicating that Drp1 knockdown suppresses NF-κB/NLRP3 signaling in MSU-stimulated macrophages. These data indicate that Drp1-increased mitochondrial ROS activated NF-κB pathway and NLRP3 inflammasome, thus inducing gout arthritis.

Several types of post-translational modifications of Drp1 have been reported, including SUMOylation, O-GlcNAcylation, S-nitrosylation, phosphorylation and ubiquitination [35]. Previous study has demonstrated that inhibition of ubiquitination and proteasomal degradation of Drp1 causes uneven distribution of mitochondria during mitosis, as well as incomplete cytokinesis. APC/C(Cdh1) has been identified as an E3 ubiquitin ligase complex responsible for Drp1 stability [36]. In the current study, USP16 was identified as a DUB which mediated deubiquitination and stabilization of Drp1 in macrophages. Co-IP and GST pull-down assay confirmed the direct association between USP16 and Drp1. Loss of USP16 promoted the degradation of Drp1 via ubiquitin–proteasome pathway, suggesting that USP16-dependent deubiquitination plays an indispensable role in maintaining Drp1 stability. Loss-of function studies further revealed the critical role of USP16 in mitochondrial fission and activation of NF-κB/NLRP3 signaling. A recent study has illustrated that USP16 functions as a key regulator of IKKβ ubiquitination and modulates NF-κB p105 phosphorylation, thus modulating NF-κB signaling and CRC tumorigenesis [20]. Besides the direct regulation of NF-κB signaling, our results demonstrated that USP16 also activated NF-κB signaling via Drp1-dependent mitochondrial fission in gout arthritis. These findings broaden the understanding of Drp1 ubiquitination by identifying the novel DUB USP16.

## Conclusion

In conclusion, we found that DUB USP16 induced gouty arthritis via Drp1-dependent mitochondrial fission and NF-κB/NLRP3 signaling. Our study provides profound implications for therapeutic strategies of gouty arthritis.

## Supplementary Information

**Additional file 1:** **Figure S1.** Validation of knockdown andoverexpression experiments. (A) The knockdownefficiency of sh-Drp1 in RAW264.7 and J774A.1 cells was detected by western blot. (B) The overexpressionof Drp1 RAW264.7 and J774A.1 cellswas detected by western blot. (C) The knockdownefficiency of sh-USP16 in RAW264.7 and J774A.1 cells was detected by western blot. *,*P* < 0.05, **, *P* < 0.01, ***, *P* <0.001.

**Additional file 2:** **Figure S2.** Drp1 regulates NLRP3inflammasome via NF-κB signaling. The protein levels of Drp1and key molecules of NF-κB/NLRP3 signaling in RAW264.7 and J774A.1 cells were detected by western blot. *, *P*< 0.05, **, *P* < 0.01.

**Additional file 3:** **Figure S3.** Mitochondrial ROScontributes to Drp1-induced NLPR3 inflammasome activation. (A) The proteinlevels of NLRP3 and Caspase-1 in RAW264.7 and J774A.1 cells were detected bywestern blot. (B) The protein levels of NLRP3 and Caspase-1 inMSU-stimulated RAW264.7 and J774A.1 cells were detected by westernblot. *, *P* < 0.05, **, *P* <0.01, ***, *P* < 0.001.

## Data Availability

Data sharing not applicable to this article as no datasets were generated or analysed during the current study.

## Abbreviations

AKI: Acute kidney injury
ATCC: American Type Culture Collection
CAT: Catalase
CHX: Cycloheximide
Co-IP: Co-immunoprecipitation
CRC: Colorectal cancer
Drp1: Dynamin-related protein 1
DUB: Deubiquitinase
SH-Px: Glutathione peroxidase
H&E: Hematoxylin & eosin
IHC: Immunohistochemistry
IL-1β: Interleukin-1β
I/R: Ischemia–reperfusion
LDH: Lactate dehydrogenase
MFI: Mean fluorescence intensity
MSU: Monosodium urate
NLRP3: Nucleotide-binding oligomerization domain-like receptor containing pyrin domain 3
PFA: Paraformaldehyde
ROS: Reactive oxygen species
SOD: Superoxide dismutase
TEM: Transmission electron microscopy
USP16: Ubiquitin specific peptidase 16

## References

[1] So AK, Martinon F (2017). Inflammation in gout: mechanisms and therapeutic targets. Nat Rev Rheumatol.

[2] Singh JA, Gaffo A (2020). Gout epidemiology and comorbidities. Semin Arthritis Rheum.

[3] Kingsbury SR, Conaghan PG, McDermott MF (2011). The role of the NLRP3 inflammasome in gout. J Inflamm Res.

[4] Broz P, Dixit VM (2016). Inflammasomes: mechanism of assembly, regulation and signalling. Nat Rev Immunol.

[5] Pazar B (2011). Basic calcium phosphate crystals induce monocyte/macrophage IL-1beta secretion through the NLRP3 inflammasome in vitro. J Immunol.

[6] Dan Dunn J (2015). Reactive oxygen species and mitochondria: A nexus of cellular homeostasis. Redox Biol.

[7] Han Y (2018). Reactive oxygen species promote tubular injury in diabetic nephropathy: The role of the mitochondrial ros-txnip-nlrp3 biological axis. Redox Biol.

[8] Kraus F (2021). Function and regulation of the divisome for mitochondrial fission. Nature.

[9] Park S (2015). Defective mitochondrial fission augments NLRP3 inflammasome activation. Sci Rep.

[10] Zhou K (2017). RIP1-RIP3-DRP1 pathway regulates NLRP3 inflammasome activation following subarachnoid hemorrhage. Exp Neurol.

[11] Liu R (2020). An Inhibitor of DRP1 (Mdivi-1) Alleviates LPS-Induced Septic AKI by Inhibiting NLRP3 Inflammasome Activation. Biomed Res Int.

[12] Qin Y (2021). Atractylenolide I Inhibits NLRP3 Inflammasome Activation in Colitis-Associated Colorectal Cancer via Suppressing Drp1-Mediated Mitochondrial Fission. Front Pharmacol.

[13] Park J (2013). Mitochondrial dynamics modulate the expression of pro-inflammatory mediators in microglial cells. J Neurochem.

[14] Liu X (2022). Mdivi-1 Modulates Macrophage/Microglial Polarization in Mice with EAE via the Inhibition of the TLR2/4-GSK3beta-NF-kappaB Inflammatory Signaling Axis. Mol Neurobiol.

[15] Qiao Y (2012). TLR-induced NF-kappaB activation regulates NLRP3 expression in murine macrophages. FEBS Lett.

[16] Bednash JS, Mallampalli RK (2016). Regulation of inflammasomes by ubiquitination. Cell Mol Immunol.

[17] Mevissen TET, Komander D (2017). Mechanisms of Deubiquitinase Specificity and Regulation. Annu Rev Biochem.

[18] Joo HY (2007). Regulation of cell cycle progression and gene expression by H2A deubiquitination. Nature.

[19] Yang W (2014). The histone H2A deubiquitinase Usp16 regulates embryonic stem cell gene expression and lineage commitment. Nat Commun.

[20] Yu, J.S., et al., Substrate-specific recognition of IKKs mediated by USP16 facilitates autoimmune inflammation. Sci Adv, 2021. **7**(3). 10.1126/sciadv.abc4009 PMC7806237 33523871

[21] Xu H (2023). Type II collagen facilitates gouty arthritis by regulating MSU crystallisation and inflammatory cell recruitment. Ann Rheum Dis.

[22] He H (2018). Oridonin is a covalent NLRP3 inhibitor with strong anti-inflammasome activity. Nat Commun.

[23] Li ZL (2021). Naringin improves sepsis-induced intestinal injury by modulating macrophage polarization via PPARgamma/miR-21 axis. Mol Ther Nucleic Acids.

[24] Kao, S.H., et al., Analysis of Protein Stability by the Cycloheximide Chase Assay. Bio Protoc, 2015. 5(1). 10.21769/BioProtoc.1374 PMC5659619 29082276

[25] Huang Q (2020). HSP60 Regulates Monosodium Urate Crystal-Induced Inflammation by Activating the TLR4-NF-kappaB-MyD88 Signaling Pathway and Disrupting Mitochondrial Function. Oxid Med Cell Longev.

[26] Brixner DI. and MJ Ho, Clinical, humanistic, and economic outcomes of gout. Am J Manag Care, 2005. 11(15 Suppl): S459–64; quiz S465–8. 16300460

[27] Dehlin M, Jacobsson L, Roddy E (2020). Global epidemiology of gout: prevalence, incidence, treatment patterns and risk factors. Nat Rev Rheumatol.

[28] Friedman JR (2011). ER tubules mark sites of mitochondrial division. Science.

[29] Zaja I (2014). Cdk1, PKCdelta and calcineurin-mediated Drp1 pathway contributes to mitochondrial fission-induced cardiomyocyte death. Biochem Biophys Res Commun.

[30] Duan C (2020). Activated Drp1-mediated mitochondrial ROS influence the gut microbiome and intestinal barrier after hemorrhagic shock. Aging (Albany NY).

[31] Sorbara MT, Girardin SE (2011). Mitochondrial ROS fuel the inflammasome. Cell Res.

[32] Abais JM (2015). Redox regulation of NLRP3 inflammasomes: ROS as trigger or effector?. Antioxid Redox Signal.

[33] Latz E, Xiao TS, Stutz A (2013). Activation and regulation of the inflammasomes. Nat Rev Immunol.

[34] Chen CJ (2006). MyD88-dependent IL-1 receptor signaling is essential for gouty inflammation stimulated by monosodium urate crystals. J Clin Invest.

[35] Hu, C., Y. Huang, and L. Li, Drp1-Dependent Mitochondrial Fission Plays Critical Roles in Physiological and Pathological Progresses in Mammals. Int J Mol Sci, 2017. 18(1). 10.3390/ijms18010144 PMC5297777 28098754

[36] Horn SR (2011). Regulation of mitochondrial morphology by APC/CCdh1-mediated control of Drp1 stability. Mol Biol Cell.

