# Supplementary figures and images for: Deubiquitinase USP16 induces gouty arthritis via Drp1-dependent mitochondrial fission and NLRP3 inflammasome activation
Source: Arthritis Res Ther. 2023 Jul 24;25:126. doi: 10.1186/s13075-023-03095-7 (PMC10367261; doi:10.1186/s13075-023-03095-7)

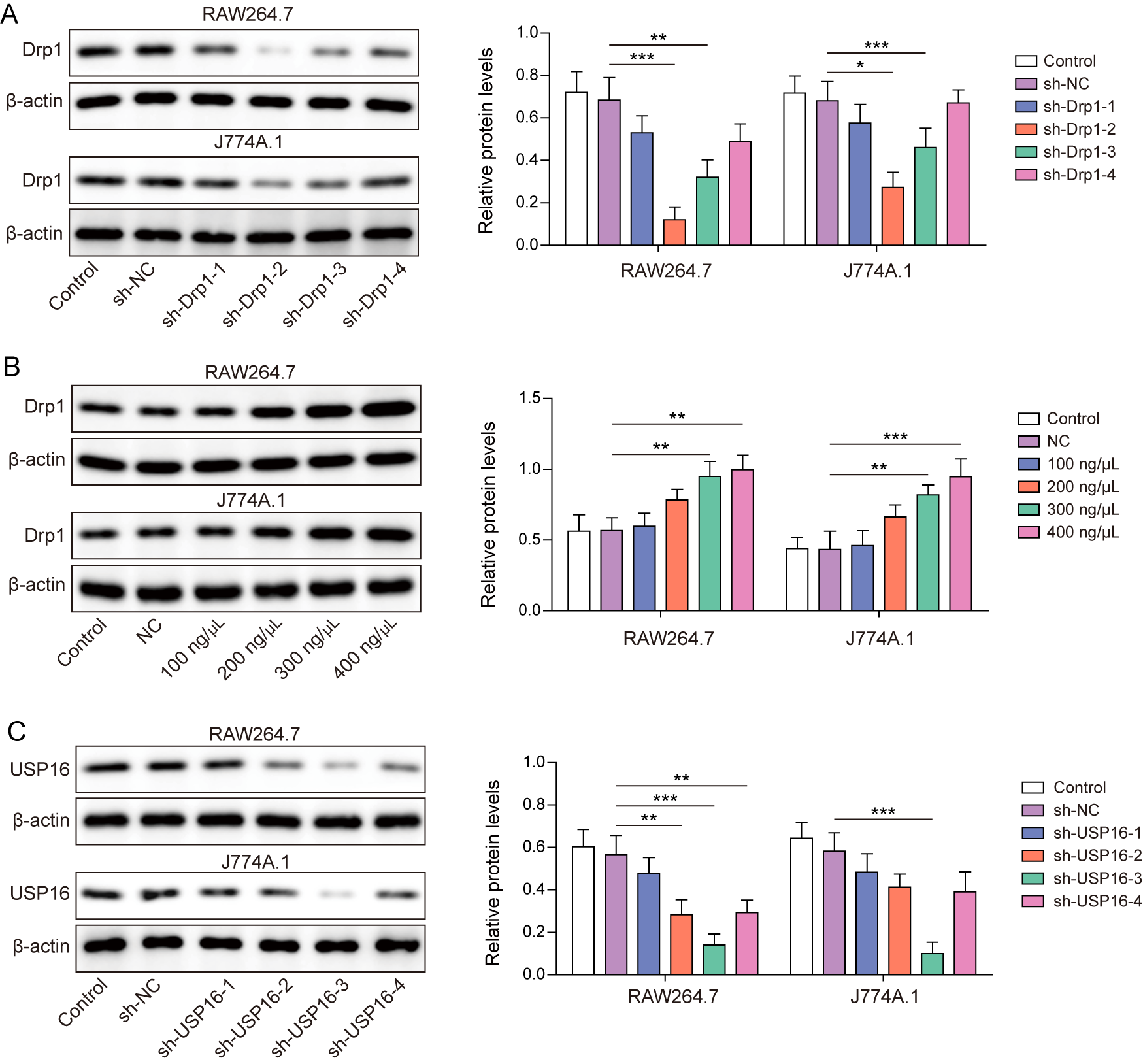

Supplement: Supplementary file 1 — Additional file 1: Figure S1. Validation of knockdown andoverexpression experiments. (A) The knockdownefficiency of sh-Drp1 in RAW264.7 and J774A.1 cells was detected by western blot. (B) The overexpressionof Drp1 RAW264.7 and J774A.1 cellswas detected by western blot. (C) The knockdownefficiency of sh-USP16 in RAW264.7 and J774A.1 cells was detected by western blot. *,P < 0.05, **, P < 0.01, ***, P <0.001. [file 13075_2023_3095_MOESM1_ESM.tif]

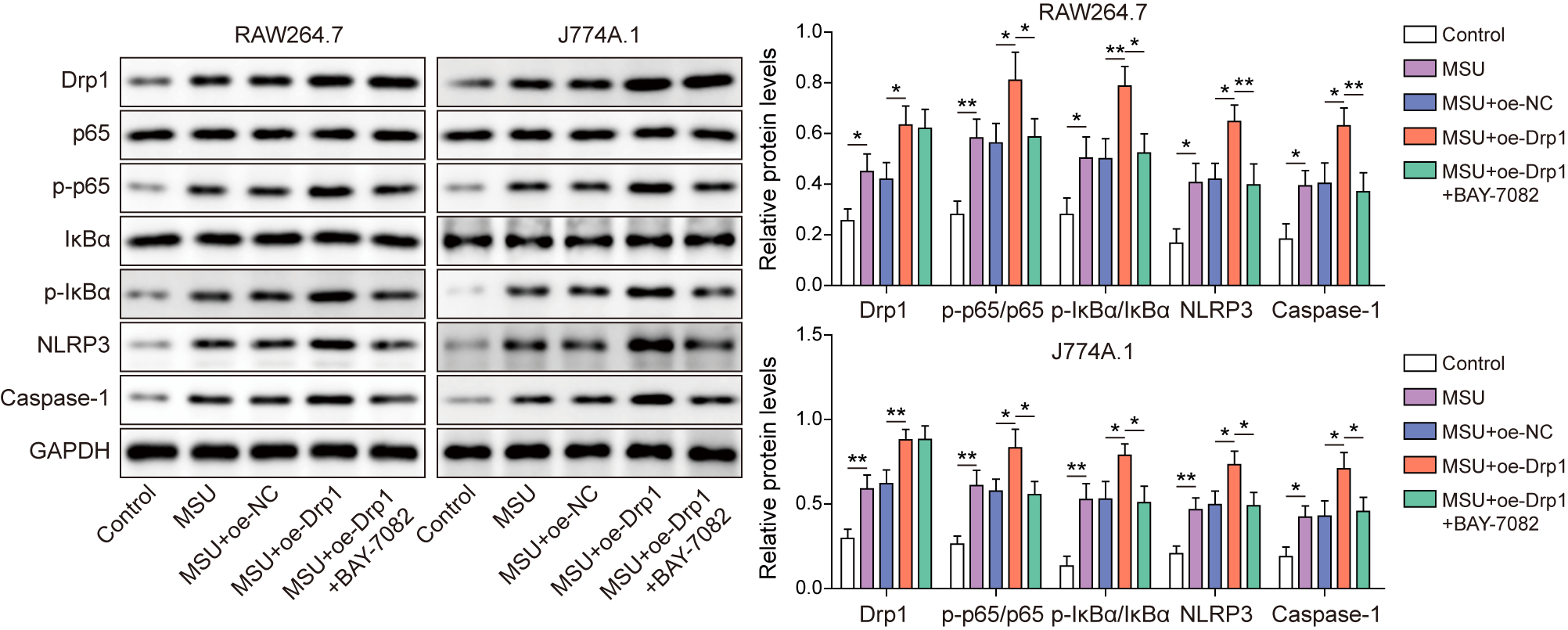

Supplement: Supplementary file 2 — Additional file 2: Figure S2. Drp1 regulates NLRP3inflammasome via NF-κB signaling. The protein levels of Drp1and key molecules of NF-κB/NLRP3 signaling in RAW264.7 and J774A.1 cells were detected by western blot. *, P< 0.05, **, P < 0.01. [file 13075_2023_3095_MOESM2_ESM.tif]

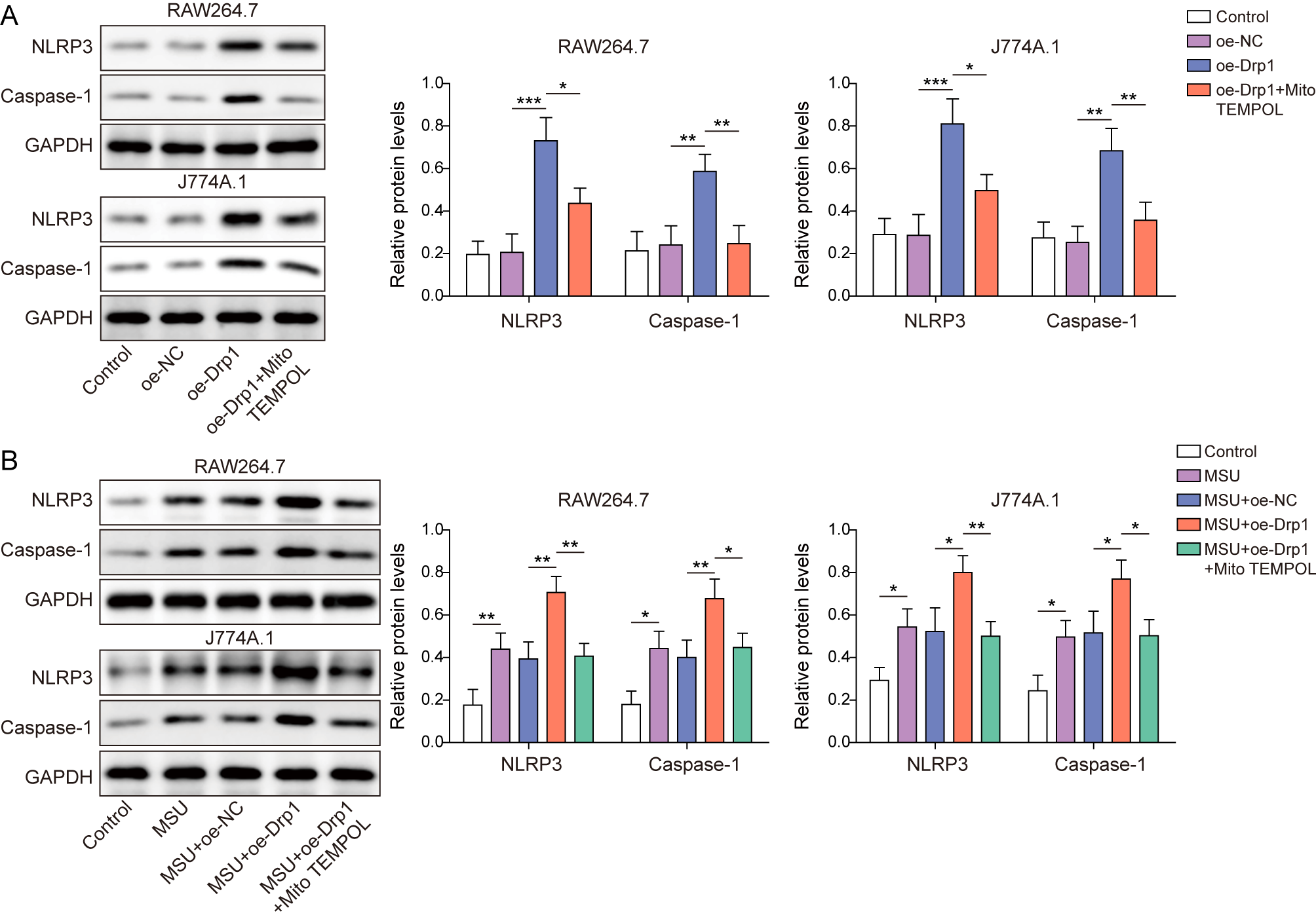

Supplement: Supplementary file 3 — Additional file 3: Figure S3. Mitochondrial ROScontributes to Drp1-induced NLPR3 inflammasome activation. (A) The proteinlevels of NLRP3 and Caspase-1 in RAW264.7 and J774A.1 cells were detected bywestern blot. (B) The protein levels of NLRP3 and Caspase-1 inMSU-stimulated RAW264.7 and J774A.1 cells were detected by westernblot. *, P < 0.05, **, P <0.01, ***, P < 0.001. [file 13075_2023_3095_MOESM3_ESM.tif]
